# Polarography Can Successfully Quantify Heavy Metals in Dentistry

**DOI:** 10.3390/medicina58030448

**Published:** 2022-03-20

**Authors:** Bahareh Nazemisalman, Narges Bayat, Shayan Darvish, Saeedeh Nahavandi, Mehran Mohseni, Ionut Luchian

**Affiliations:** 1 Department of Pediatric Dentistry, School of Dentistry, Zanjan University of Medical Sciences, Zanjan 4513956184, Iran; drb.nazemi@zums.ac; 2 School of Dentistry, Zanjan University of Medical Sciences, Zanjan 4513956184, Iran; nargesbayat91@gmail.com; 3 Department of Oral and Maxillofacial Radiology, School of Dentistry, Tabriz University of Medical Sciences, Tabriz 5166614766, Iran; 4 Pardis Health Center, Shahid Beheshti University of Medical Sciences, Tehran 1658133841, Iran; shayand@dentistry.ubc.ca; 5 Faculty of Dentistry, University of British Columbia, Vancouver, BC V6T 1Z3, Canada; 6 Food and Drug Control Department, Zanjan University of Medical Sciences, Zanjan 4513956184, Iran; nahavsn@gmail.com; 7 Department of Periodontology, Faculty of Dental Medicine, “Grigore T. Popa” University of Medicine and Pharmacy, Iasi 700115, Romania; ionut.luchian@umfiasi.ro

**Keywords:** polarography, heavy metals, dentistry, teeth, copper, cadmium, lead

## Abstract

*Background and Objectives*: Due to the nutritional and behavioral patterns of children, their teeth can be a good indicator of heavy metal uptake from over the years. To determine the amount of Zn, Cu, Cd, and Pb accumulated in the body, primary teeth of children in Zanjan, Iran, were examined with a polarography device. *Materials and Methods*: Samples were collected from dentistry clinics of Zanjan, Iran, and were prepared for acid digestion, and then were analyzed by a polarography device for determining the concentration of lead, copper, zinc, and cadmium. *Results*: Data were analyzed by a t-independent test to compare different groups (*p* < 0.05). Based on the results obtained from this study, the mean concentrations of zinc, lead, copper, and cadmium were 245, 7.66, 5.33, and 0.0879 µg/g, respectively, which shows that the amount of each of the four elements was more than the amounts that have been reported for different countries. The results showed no significant difference between age, tooth type, and jaw groups. *Conclusions*: We conclude that primary teeth are an important biological indicator to evaluate the concentration of heavy elements in the human body. The high concentrations of these elements in the primary teeth analyzed in this study could be attributed to the high concentrations of these elements in the environment of Zanjan.

## 1. Introduction

Human societies and the surrounding environment have been exposed to high levels of heavy elements because of industrial progress and as a result of actions such as modern agriculture, urbanization, and the use and distribution of underground resources [1,2]. These elements can become part of the body and can accumulate in both structural tissues such as bones, hair, nails, and teeth, and in vital organs such as the kidney, brain, and liver during biological activity, such as breathing, digestion, or even skin contact, thus influencing the health of humans and causing tremendous danger in vital organs [3,4]. Children are more susceptible to being exposed to heavy metals than adults due to their different dietary behaviors and lifestyles. As an example, for a child, the breathing zone is closer to the floor due to the height and mobility of the child [5].

Lead is one of the most prevalent toxic metals humans are exposed to [6,7]. After exposure, lead is predominantly stored in bone, and therefore teeth have been widely used to evaluate environmental lead exposure [6,8,9,10,11,12]. The levels of Pb in teeth are influenced by factors such as the age, teeth type, geographic location, and economic status of individuals [8,9]. As the age increases, the amount of lead in dentin and dentinal pulp increases [13,14]. However, according to previous studies, gender does not cause changes in lead accumulation [4,10]. There is no physiological function for lead and cadmium, and they are harmful to the body even in small quantities [8]. These two elements mainly contaminate air and food, and enter the body through breathing contaminated air and eating food [15]. On the other hand, zinc and copper are essential elements for the normal function of the body, and play an important role in the formation and metabolism of mineral tissues [16,17]. However, excessive levels of zinc and copper in the body are toxic and can produce respiratory issues, stomach toxicity, and anemia, and can cause damage to the kidneys and digestive tract, coma, death, and Wilson’s disease, resp8ectively [16]. It is important to consider that the deficiency of this element is as dangerous as its excessive increase [15]. Hair, nails, and teeth can not only accumulate toxic elements such as lead and cadmium, but also essential elements such as nickel, chromium, zinc, sodium, manganese, calcium, copper, potassium, fluoride, and cobalt. Therefore, they can be useful for the determination of environmental pollution [18]. Because the hard tissue of the tooth can accumulate metals and due to the ease of collecting teeth during shedding, many research studies have covered the presence of heavy metals in the teeth [15,19]. To do this in our study, a polarographic device was used to evaluate the concentration of elements because of its cost-effectiveness and less time spent on most of the analytical techniques, despite the high technique sensitivity [20,21]. Various methods have been proposed to identify metal ion shapes. The polarography method has been introduced as one of the most common methods. In general, polarography is a type of voltammetry where the working electrode is a dropping mercury electrode (DME) to monitor changes in the current upon the application of a voltage. In this technique, oxidation and reduction of the current flowing from a cell occur based on the working electrode potential. In truth, metal ions, e.g., Pb and Cd ions, are reduced inside the polarography cell by applying potential, and are dissolved inside the mercury. The reasons for the superiority of this technique over other methods can be attributed to the wide range of traceable concentrations and measurable inorganic and organic substances and organic metals in the PPB scale. Some advantages of this method over many other common techniques include wider linear ranges and analysis of a variety of soluble and solid samples. Other advantages of the polarography technique include simultaneously measuring several metals in a sample, thus decreasing the analytical costs compared to the atomic absorption method, as well as the simplicity of the operation [22].

Considering what has been explained about the effects and ways of transmission of these elements, by knowing that the largest lead and zinc mine is in Zanjan province, and also considering that studies have identified primary dentition as a suitable biological indicator for environmental pollution [18], we decided to determine the concentration of copper, zinc, cadmium, and lead in the teeth of children living in Zanjan Province.

## 2. Materials and Methods

### 2.1. Sample Collection and Preparation

The chosen samples were composed of 96 deciduous teeth of different sexes and ages of children with high economic levels living in Zanjan, far from the source of heavy metals such as specific factories, which were extracted due to their dental treatment plan determined by their dentist. The carious lesion, because of not being sure about the different concentrations of metals in the sound and carious teeth, would be removed up to 2 mm deeper than the surface of the carious lesion to certainly eliminate the effect of an active lesion on the mineral content of dentine, and the pulp of all teeth were removed with endodontic files [23]. Teeth with amalgam fillings and those that were near the teeth with fillings were excluded from the study. All of the samples were grouped based on tooth type (anterior and posterior), tooth position within the mouth (upper and lower jaw), age, and sex. 

To clean the samples, all of the teeth were cleaned with doubly distilled water (DDW) and were then immersed in 10% HNO_3_ overnight and washed several times with DDW, and finally were rinsed with deionized water [24] before analyzing and removing the materials deposited on the surface of the samples, such as dental plaque or adipose tissue. Each tooth was immersed in 30% H_2_O_2_ for 2 h and was thoroughly washed with deionizing water several times. Samples were placed in an oven at 50 °C for 24 h to be completely dry and were then weighted. It was impossible to prepare samples with the actual same weight (0.5 g). The sample’s real weight (0.45–0.55 g) was applied for digestion, but the correction coefficient was used for reporting the final results. Finally, each sample with a range of 0.45–0.55 g weight was digested in a Teflon vessel, adding 6 mL of HNO_3_ 65%, 3 mL of HF 40%, and 2 mL of H_2_O_2_ 30% using a Sineo Microwave Digestion device (MDS-10, China). One vessel was used as a blank, containing reagents without samples, for validation of the measurement. All reagents used in this procedure were of the highest obtainable purity (supra pure), and were purchased from Merck, Germany. Digestion was done during four heat steps, with the same power for each vessel as listed in Table 1. Digested samples were kept in the refrigerator in separate test tubes until measuring with a polarography instrument, as there were different.

### 2.2. Device Specifications

A polarographic system (Model 797 VA, Metrohm, Switzerland) equipped with a working electrode (MME; model 6/1246/020), reference electrode (Ag/AgCl; model 6/0728/020), auxiliary electrode (platinum rod; model 60,003/343,000), nitrogen (99.99%), pure gas, nitrogen regulator with outlet pressure rating, and mercury with a purity of 99.99% (Merck CAS Number = 4403) was employed. According to application bulletin no. 231/2 and DIN 38,406 part 16 for measuring copper, zinc, cadmium, and lead by polarography instrument, digested samples were prepared according to the device instructions, and the concentrations of copper, zinc, cadmium, and lead were measured three times via the ASV (Anodic Stripping Voltammetry) method. The working electrode was the hanging mercury drop electrode (HMDE); the stirring rate (stirrer/RDE) was 2000 rpm; measurement mode was DP (differential pulse); aeration time by nitrogen for deoxygenating of the solution (purge time) was 300 s; deposition potential was −1.15 V; deposition time was 90 s; the start and end potentials were −1.15 and 0.05 V; and the anodic potentials were −0.98, −0.56, −0.38, and −0.10 V for zinc, cadmium, lead, and copper, respectively. Lead, cadmium, zinc, and copper concentrations in the sample were measured with two duplicates. As this method uses standard addition for calibration, 0.1 mL of standard solutions of lead, cadmium, zinc, and copper were added simultaneously to the cell containing the sample, and the above steps were repeated. For the second time, standard solutions were added to the polarography cells, and the measurements were repeated. In addition, polarograms were drawn during each assay, in which vertical and horizontal axes represented the current intensity and potential difference, respectively. Eventually, reports were presented by the device following completing each experiment, and the concentrations were determined in µg/L (24). It should be noted that the detectable range of copper and zinc in the polarography device was 1 μg/L–50 mg/L, and for cadmium and lead was 0.1 μg/L–50 mg/L.

### 2.3. Quality Controls of the Method

In this study, values of the limit of detection (LOD) and the limit of quantification (LOQ) were also determined for lead, cadmium, copper, and zinc metals to evaluate the validation of the test method with suitable levels of accuracy and precision. To calculate the limit of detection (LOD) in terms of analyte concentration, a signal equal to the blank signal (yB) was utilized, plus three standard deviations (sB) of the blank signal, (yB + 3sB). Additionally, the quantitation limit (LOQ) was considered to be the lowest limit for precise quantitative measurements, as opposed to qualitative detection. To achieve this limit, a value of yB+10sB was used [25].

In addition, standard concentrations of 25, 50, 100, and 200 µg/L were prepared from the standard solutions of lead, cadmium, copper, and zinc to evaluate the accuracy and precision of the method, and their values were measured by the device. Ultimately, the resulting relative standard deviations (RSDs) were investigated.

#### Standard Reference Materials

Standard stock solution concentrations were 10, 0.5, 0.1, and 2.5 mg/L for zinc, lead, cadmium, and copper, respectively, which were prepared from Merck Titrisol stocks (Zn(NO_3_)_2_ in HNO_3_ 0.5 mol/l 1000 mg/L, Pb(NO_3_)_2_ in HNO_3_ 0.5 mol/L 1000 mg/L, Cd(NO_3_)_2_ in HNO_3_ 0.5 mol/L, 1000 mg/L, and Cu(NO_3_)_2_ in HNO_3_ 0.5 mol/l 1000 mg/L) and were diluted with HNO_3_ 0.014 mol/L solution. 

### 2.4. Sample Analysis

**Figure 1:**
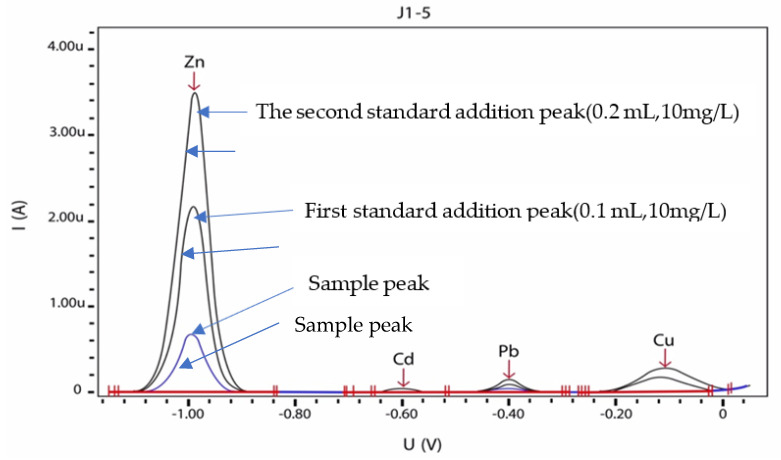
Determination of zinc, cadmium, lead, and copper in sample No. J1-5 and its polarographic curves according to DIN38406/16. Spiked concentrations were 0.1 mL of 10, 0.5, 0.1, and 2.5 mg/L of zinc, lead, cadmium, and copper, respectively.

Concentrations of copper, zinc, cadmium, and lead in each digested tooth were found by using 797 VA Computrace software. For the analysis, standard solutions of zinc, copper, lead, and cadmium were prepared as mentioned previously. For preparing the KCl-sodium acetate solution, 55.9 g KCl (CAS 7447-40-7) + 25 mL NaOH 30% (CAS 1310-73-2) + 14.2 mL CH3COOH 100%(CAS 64-19-7) were filled up to 500 mL with ultrapure water. Then, 10 milliliters of the specimen were adjusted to pH 4.6 by 1 milliliter of KCl-sodium acetate solution in a polarography cell, and the concentration of the desired metals was obtained by adding the standard addition. After preparing the samples and injecting them and applying the necessary instructions to the system, eventually, the presence of each of the metals was shown as a peak on the scale (μg/g) by the device’s software report. In this article, 96 dental samples were collected, but 20 samples were removed before the digestion due to a lack of adequate weight after removing dental plaque or adipose tissue, and 34 of the remaining sample concentrations were less than the LOD of the method for the elements, and were thus omitted. Only 42 samples were reported in this research. Figure 1 shows a sample of polarographic curves given by the polarography device. Blueline shows the sample curve of No. J1-5 in the potential voltage of zinc, Cd, Pb, and Cu; black lines are two replications of the standards amount of zinc, Cd, Pb, and Cu in potential voltage of zinc, Cd, Pb, and Cu. The *X*-axis shows the anodic potentials of zinc, cadmium, lead, and copper, respectively in Volte. The *Y*-axis shows the current intensity, which is related to the concentration of samples in Ampere. 

Each dental specimen digestion was repeated three times and, in each case, “mean ± SD” was calculated and the results were compared with a t-independent test using SPSS software 19. We used a one-sample Kolmogorov–Smirnov test to evaluate the normality of the metals concentration and noticed that all of them were normally distributed. In addition, we used an independent sample t-test to compare the metal concentration according to gender, age group, jaws, and teeth.

## 3. Results

### 3.1. LOD, LOQ, Accuracy, and Precision

The signals from 10 blank injections into the polarograph at specific potentials for zinc, copper, lead, and cadmium were obtained. The regression coefficient for zinc was 0.9987 and the LOD and LOQ values were evaluated equal to 0.317 and 0.889 ppb, respectively. The regression coefficient for copper was 0.9995 and the LOD and LOQ values were evaluated equal to 0.286 and 0.943 ppb, respectively. The regression coefficient for the lead was 0.9985 and the LOD and LOQ values were evaluated equal to 0.281 and 0.936 ppb, respectively. The regression coefficient for cadmium was 0.9989 and the LOD and LOQ values were evaluated to be equal to 0.261 and 0.871 ppb, respectively.

As shown in Table 2, in the accuracy test, it was observed that the data were very close to the actual value and were in the optimal range of 94 to 106%. In terms of precision, RSD was less than 5%.

### 3.2. Results of Samples Masurment

**Figure 2:**
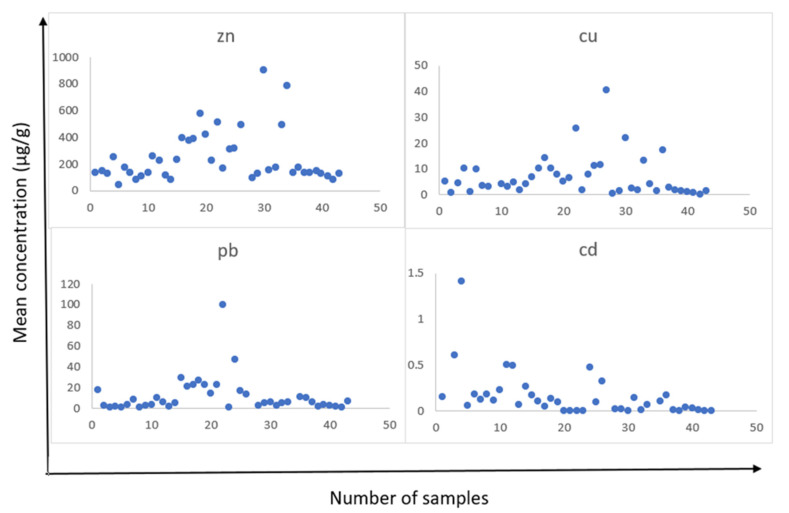
The distribution of zinc, Cd, Pb, and Cu concentrations in each numbered sample after three replications.

**Figure 3:**
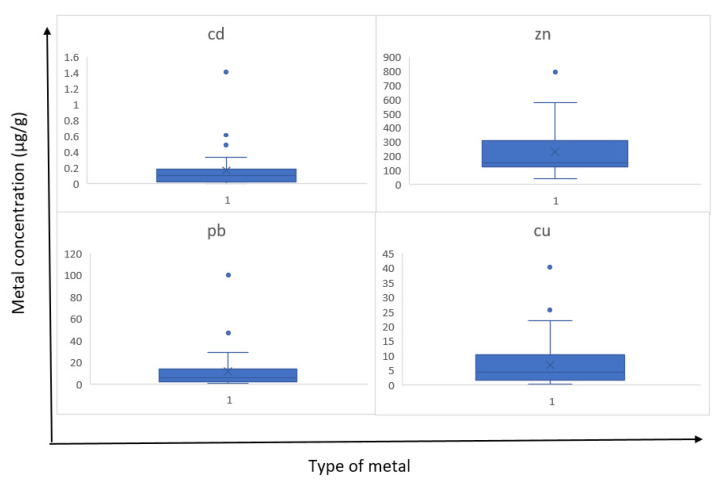
Boxplot of Zn, Cd, Pb, and Cu concentrations in the samples. The minimum (0th percentile), maximum (100th percentile), median (50th percentile), first quartile (25th percentile), and third quartile (75th percentile), clearly show the data scatter in the first, second, third, and fourth quartiles. The most dispersion was shown in the fourth quartiles.

Heavy metals are increasingly contaminating the environment and play an important role in the development of human disease and have toxic effects. Increasing the prevalence of these metals, as a result of natural and human processes, can lead to widespread clinical effects [4,20]. In addition, studies have already shown that the amount of elements in the body varies with the environment around people and for their concentration, and dental teeth can be good biological indicators [26]. From the results of a total of 42 people, there were 24 girls and 18 boys aged 2.5 to 8 years with an average of 5.6 years, with a mean dental weight of 0.429 g. The results obtained from the amount of lead, zinc, copper, and cadmium elements are summarized in Table 3. According to this table, among the four elements studied, the order of the average concentration of the elements was as follows: Zn > Pb > Cu > Cd. The distribution of concentration of these elements in each sample is shown in Figure 2. As seen in the boxplots, in Figure 3, although the focus was mostly on the average points, data dispersion were evident in points above the mean and even the fourth quartile, the area between the third quartile and maximum. The results obtained about varying concentrations of heavy metals among different groups of tooth type, tooth position, sex, and jaw are summarized in Table 4.

According to this, although the mean of these four elements were not so high, all of them had large dispersions and coefficient of variations (CV%). CV is the ratio of the standard deviation to the mean, and the higher the coefficient of variation, the greater the level of dispersion around the mean. Considering the high doses of these metals in the body, it is important to pay attention to those who are in the third quartile in Figure 3. This high amount of metal could be attributed to the economic status of people, their nutritional behavior, and their place of residence.

## 4. Discussion

Copper and zinc are both important and vital elements in humans, but their high level of concentration can have many destructive effects on the human body [24]. Increased copper can cause anemia, allergy, autism, increased adrenal function, hair loss, headache, depression, bone fracture, dental caries, heart attack, infections, and even cancer in the human body [27]. Increased zinc can also be due to a disorder in copper and iron absorption, and can lead to impairment of cellular function, which causes the death of cells, of which the main organ is the brain [28].

Excluding 491 µg/g as the outlier data among the results obtained for the amounts of lead in the samples, concerning the concentration of lead, the minimum and maximum levels of 0.6 μg/g and 22.80 μg/g were achieved with a mean of 7.66 μg/g, which is ranked second in terms of dispersion. Lead is one of the most common toxic substances in the world, and is considered as an environmental pollutant due to industrialization. Lead enters the food cycle through the contamination of soil, food, water, and air, thus causing dangerous effects on human health [2,16].

This metal is also considered a toxic element, which, even at low levels, has devastating effects on the body’s organs [16,29]. Exposure to low doses over a long time may cause destructive effects to occur [30]. This element can impair the amylogenesis in the developmental process of teeth, and its presence in enamel is an important indicator of the presence of this element in the surrounding environment [31,32].

There are also plenty of studies about varying concentrations of heavy metals among different groups of tooth type, tooth position, sex, and age, but their results are somehow in contrast. The results obtained from this article is summarized in Table 4, and here we discuss the differences among groups.

### 4.1. Gender

Although the mechanisms are unclear, studies of metal exposure show that women often have higher blood and hair levels of metals than men in the same families or communities [33]. Previous studies, including Bayo. j et al. [34] and Alomary A. et al. [4], showed that the concentration of cadmium in boys and girls was not different, but Alomary reported a significant difference in lead level between the teeth of the boys and girls. In our study (according to Table 4), the concentration of lead, copper, and zinc in girls was more than that in boys, but just the amount of zinc was significantly higher (*p* < 0.05). In addition, the result reported from the study of Asaduzzaman et al. in 2017 showed more heavy elements in girls than boys [29], but on the other hand, some articles, such as Pashmi and Pour Khabaraz in 2012, reported a higher concentration of copper in boys because of the long-term exposure of boys to contaminants in the environment, while a significant difference in zinc level between the teeth of the boys and girls was not reported [27]. The existence of extensive zinc mines in Zanjan province may cause significantly different amounts of zinc compared to other metals, which may increase the presence of zinc in water and food sources and its accumulation in deciduous teeth. In addition, high levels of zinc in males may be due to physiological differences between males and females.

The differences between the findings of this study and other researchers can be due to various reasons, including differences in the sample size, measurement method, and age of individuals. The sample size in Alomary, Bayo, Asaduzzaman, and Pashmi was 268, 371, 50 and 108, respectively. Alomary and Pashmi applied atomic absorption spectroscopy, while Bayo and Asaduzzaman used anodic stripping voltammetry and inductively coupled plasma mass spectrometry, respectively. The Alomary study, unlike other research, was performed on adult teeth.

### 4.2. Age

The results of the study indicate that all metals are slightly elevated in older children, but not statistically significantly. However, the results of other studies about the influence of age are in contrast, for example in studies like Bayo [34], concentrations of cadmium and lead; Kern and Mathiason [35], lead concentrations; and Asaduzzaman et al. [29], the concentration of the 10 heavy substances was age-dependent, while Alomary et al. [24] did not show a significant difference of heavy metal concentration until the age of 50. On the other hand, some studies reported that the concentrations of copper and zinc were statistically different depending on the type of teeth [27].

The results of Kern, Asaduzzaman, and Alomary were obtained from people over the age of 10, 15, and 20 years, respectively. Bayo devoted a part of their study to the teeth of children under 10 years old. All researchers pointed out that after the age of 10, the amount of lead and cadmium in permanent teeth increases with age, which is not comparable to the present study due to age differences. Bayo showed that with increasing age up to 10 years old, the amount of lead and cadmium in deciduous teeth decreases, which is not consistent with the present study, which may be due to differences in sample size, measurement method, or the proximity of people in the Zanjan province to lead and zinc mines.

### 4.3. Tooth Type

Although the amounts of zinc, cadmium, and lead in incisors are higher and the copper is lower than molars in the present study, no significant difference was found between tooth types. However, there was no agreement on the increase or decrease of metal concentration through the incisors towards the molars. Past studies like Asaduzzaman et al. [29], Bayo et al. [34], and Tvinnereim et al. [8] showed that the amount of heavy metals differ according to the type of teeth, and some elements such as mercury, copper, and tin in the molars and lead, strontium, antimony, and zinc in the incisors are higher, which is consistent with the present study. As the incisors are at the front of the oral cavity, they may have more contact with suspended dust and saliva, and may cause an increase of zinc, lead, and cadmium in the incisors. The increase in zinc and lead concentration in the incisors may be a reflection of the pollution caused by industrial activities in Zanjan province. 

Although there is no evaluation about the difference between permanent and primary teeth in this study, Fischer et al. found that the concentrations of metals such as Pb and Cd were significantly higher in the deciduous teeth than in the permanent ones [36]. Other studies such as Attramadal et al. [37] reported similar levels of cadmium and zinc in deciduous and permanent teeth. The copper content varied little in deciduous teeth, but in permanent teeth, a wide variation in the copper levels was found. The content of lead in deciduous teeth was found to be higher than in the permanent teeth [37].

### 4.4. Jaw

The concentrations of zinc, cadmium, lead, and copper were 200.49, 0.98, 9.80, and 9.20 in the maxilla, and 263.14, 5.03, 28.16, and 6.89, in the mandible, respectively, however, the differences were not significant. Alomary et al. [24] reported that the concentrations of copper and cadmium in the maxilla were higher than the mandible, but the levels of lead and zinc in the two jaws were not different. Al-Haddad et al. [38] conducted a study on the concentration of cadmium, copper, and Fe, and noted that a limitation of their comparison was that the teeth were from different individuals and therefore did not reflect true differences between the upper and lower teeth.

Briefly, in this study, the mean concentrations of zinc, cadmium, lead, and copper were 245, 0.087, 7.66, and 5.33 µg/g, respectively. The concentration of zinc in this study was more than that reported by Asaduzzaman et al. [29], Tvinnereim et al. [8], and Pashmi et al. [27], who reported zinc concentrations of 3.99, 157, and 218.14 µg/g respectively. These differences could be because of various methods that each study used, for example, Asaduzzaman et al. [29] collected 50 separate human teeth from dental patients and Analysed them using inductively coupled plasma-mass spectrometry (ICP-MS). They concluded that the elevated concentration levels of heavy metals in tooth dentine reflect pollution from industrial emissions and urbanization. Tvinnereim et al. [8] analyzed 1271 samples of primary teeth using atomic absorption spectrophotometry (AAS), and concluded that metal concentrations in the primary teeth are affected by the presence of caries and roots, and by tooth group. Pashmi et al. [27] also used 108 sound primary teeth without pulp removal, and used flame atomic absorption spectrophotometry to analyze them. 

Although the concentration of lead in this study was more than that reported by Asaduzzaman et al. [29] and Tvinnereimet al. [8], who reported lead concentrations of 2.10 and 1.37 µg/g, respectively, it was lower than what was reported by Alomary et al. [24], with 30.26 µg/g. Alomary et al. [24] collected 320 deciduous teeth from 5- to 12-year-old children and analyzed them for Pb, Cd, Cu, Fe, and Zn using inductively coupled plasma–optical emission spectrometry, which could be the result of differences between the reported concentrations.

The concentration of copper in this study was more than that reported by Asaduzzaman et al. [29] and Al-Haddad et al. [38], who reported copper concentrations of 1.23 and 2.78 µg/g, respectively, but it was lower than what was reported by Pashmi et al. [27], with 8.44 µg/g. In the article of Al-Haddad et al. [38], 61 shed deciduous incisor teeth were collected from 61 children aged between 6 and 15, and they were analyzed using inductively coupled plasma mass spectrometry which is a completely different method.

The concentration of cadmium in this study was less than that reported by Alomary et al. [24] and Tvinnereim et al. [8], who which reported cadmium concentrations of 0.55 and 0.11 µg/g, respectively, but it was more than what was reported by Al-Haddad et al. [38], with 0.054 µg/g.

We also have to emphasize the limitations of the present research. The first limitation was a small sample size, due to expensive dental services and the low income of families, as well as not caring about primary teeth in under 10 year old children because they are temporary. 

The second limitation arises from the device, such as certain sample loss during the preparation process, our device detection limits, and the inability to compare different methods of digestion because of the lack of facilities.

Finally, for future studies, to reduce the limitations of this research, we considered a larger population with the participation of neighboring provinces. Providing samples from places adjacent to heavy metal mining plants, as well as places that do not have such plants or mines, may lead to more accurate statistical results. Certainly, more samples can be obtained in areas where insurance companies provide appropriate dental services.

## 5. Conclusions

According to the results of this study, although we did not have any reported threshold for the amount of heavy metals in the body, we can conclude that the amount of these heavy metals quantified at the level of teeth obtained from children living in Zanjan Province in Iran was high, which may be due to the proximity of the zinc mine. Taking into account the fact that teeth can be used as important biological indicators to measure the concentration of heavy elements in the environment, we can attribute these significantly increased values to severe pollution. Furthermore, the results of the present study demonstrate that there is not any considerable influence of tooth type, jaw, gender, and age on the Pb, Cd, Cu, and Zn content in deciduous teeth, except for the significant difference in Zn between males and females. Considering the existence and activity of lead and zinc mines in this region, it is recommended to pay more attention to the crucial influence of the living environment on general and dental health.

## Figures and Tables

**Table 1:** Properties of time, temperature, and power used for digestion of samples by a digester device “MDS-10”.

Step (N)	Temperature (Celsius)	Time (min)	Power of Single Vessels (W)
1	130	10	400
2	150	5	400
3	180	5	400
4	210	15	400

**Table 2:** The accuracy and precision of the method using the values prepared from the standard solution for zinc, copper, lead, and cadmium.

	Zn (µg/L)	Cu (µg/L)	Pb (µg/L)	Cd (µg/L)
	Mean ± SD	Mean ± SD	Mean ± SD	Mean ± SD
Actual value	200	200	200	200
Measured value	196.44 (±4.87)	200.50 (±0.619)	202.88 (±8.11)	211.01 (±9.44)
RSD%	2.46	3.08	3.99	4.47
Actual value	100	100	100	100
Measured value	99.12 (±1.22)	104.11 (±3.24)	102.07 (±1.99)	98.22 (±1.54)
RSD%	1.23	3.11	1.94	1.56
Actual value	50	50	50	50
Measured value	47.2 (±0.99)	49.9 (±1.91)	53.15 (±2.18)	50.13 (±0.65)
RSD%	2.09	3.82	4.10	1.29
Actual value	25	25	25	25
Measured value	28.33 (±0.05)	27.11(±1.10)	27.16 (±1.23)	24.18 (±0.48)
RSD%	1.76	4.05	4.52	1.98

**Table 3:** Summary statistics of heavy metal concentrations (µg/g).

	Zn	Cd	Pb	Cu
Mean	245	0.0879	7.66	5.33
N	42	42	42	42
Std. Deviation	191	0.0741	6.58	4.35
Coefficient of Variation%	77.9	84.3	85.9	81.6
Maximum	900	0.27	22.80	17.2
Minimum	40	ND *	0.60	0.30
Valuse < LOD of total sample	34 (96)	34 (96)	34 (96)	34 (96)

ND *: Not Detected.

**Table 4:** Concentration of heavy metals among different groups of tooth type, age, tooth position, and gender.

Variables			Zn (μg/g)	Cd (μg/g)	Pb (μg/g)	Cu (μg/g)
gender	Female	Mean	293.16	0.08	8.20	5.91
		Std. Deviation	225.33	0.07	6.43	4.76
	Male	Mean	181.37	0.09	6.91	4.56
		Std. Deviation	109.33	0.08	6.91	3.75
Sig. (2-tailed)			0.041	0.722	0.552	0.326
Age range	1 till 5	Mean	224.48	0.08	6.33	4.90
		Std. Deviation	181.37	0.07	5.31	4.38
	5 till 10	Mean	264.13	0.09	8.88	5.73
		Std. Deviation	202.13	0.08	7.47	4.40
Sig. (2-tailed)			0.509	0.566	0.214	0.539
Type of teeth	Incisor	Mean	311.32	0.09	8.98	5.02
		Std. Deviation	231.20	0.07	7.52	3.61
	Molar	Mean	221.80	0.08	7.20	5.44
		Std. Deviation	173.20	0.07	6.28	4.64
Sig. (2-tailed)			0.186	0.733	0.449	0.787
Type of jaw	Upper	Mean	200.60	0.06	6.79	3.88
		Std. Deviation	107.09	0.06	6.74	2.94
	Lower	Mean	263.16	0.10	8.02	5.91
		Std. Deviation	214.93	0.07	6.70	4.73
Sig. (2-tailed)			0.344	0.208	0.590	0.176

## Data Availability

All data can be provided by the corresponding author upon reasonable request.
